# Semantic Web-Based Integration of Cancer Pathways and Allele Frequency Data

**DOI:** 10.4137/cin.s1006

**Published:** 2009-01-15

**Authors:** Matthew E. Holford, Haseena Rajeevan, Hongyu Zhao, Kenneth K. Kidd, Kei-Hoi Cheung

**Affiliations:** 1 Department of Biostatistics, School of Public Health, Yale University, New Haven, CT, U.S.A; 2 Department of Genetics, School of Medicine, Yale University, New Haven, CT, U.S.A; 3 Center for Medical Informatics, School of Medicine, Yale University, New Haven, CT, U.S.A; 4 Department of Computer Science, Yale University, New Haven, CT, U.S.A

## Abstract

We demonstrate the use of Semantic Web technology to integrate the ALFRED allele frequency database and the Starpath pathway resource. The linking of population-specific genotype data with cancer-related pathway data is potentially useful given the growing interest in personalized medicine and the exploitation of pathway knowledge for cancer drug discovery. We model our data using the Web Ontology Language (OWL), drawing upon ideas from existing standard formats BioPAX for pathway data and PML for allele frequency data. We store our data within an Oracle database, using Oracle Semantic Technologies. We then query the data using Oracle’s rule-based inference engine and SPARQL-like RDF query language. The ability to perform queries across the domains of population genetics and pathways offers the potential to answer a number of cancer-related research questions. Among the possibilities is the ability to identify genetic variants which are associated with cancer pathways and whose frequency varies significantly between ethnic groups. This sort of information could be useful for designing clinical studies and for providing background data in personalized medicine. It could also assist with the interpretation of genetic analysis results such as those from genome-wide association studies.

## Introduction

Semantic technologies^1^ provide a useful means of categorizing and relating biological data. General purpose ontology languages such as OWL (Web Ontology Language) allow the biologist to create a formal structure for a knowledge domain that is both precise and logically sound.^2^ Numerous biological ontologies have been created in recent years with probably the most well-known being the Gene Ontology (GO).^3^ A major advantage of the ontological approach is that it allows us to express our data in the domain-specific terms we have defined in our ontology. This expressivity combined with logical rigor, allows us to query data based upon its meaning rather than simply how it is stored. This is generally referred to as “semantic” querying.^4^ The promise of the “Semantic Web” lies in its ability to provide links between individual semantic data stores. In this way, multiple unrelated sources of information can be queried based upon the use of commonly recognized terms. As a result, considerably more insight can be gained than from the individual data stores in isolation.^5^

The ability to query across multiple domains is especially useful in a landscape as variegated as that of biomedical informatics. Here the researcher must interact with data from a plethora of disciplines, at numerous levels of granularity and in widely divergent formats. Cancer research is further complicated by the fact that we don’t fully understand many of the mechanisms that are involved. It is to be expected then that the field of biomedical ontology has grown rapidly in recent years. Capitalizing on the success of early ontologies such as the GO^6^ and the Foundational Model of Anatomy (FMA),^7^ the Open Biomedical Ontologies (OBO) consortium now incorporates a growing collection of some 60 ontologies in an attempt to cover all areas of “biological reality”.^8^ The World Wide Web Consortium (W3C) has formed an interest group to facilitate discussion on the use of Semantic Web technology in the Health Care and Life Sciences.^9^ The National Cancer Institute’s NCI Thesaurus provides a controlled vocabulary covering a broad spectrum of topics related to all facets of cancer research.^10^ It seeks to provide a means for cancer scientists to share research in a consistent and standardized manner. The cancer Biomedical Informatics Grid (caBIG) project hopes to integrate all cancer-related research into a single massive grid, with a multi-tier semantic metadata framework to mediate between individual data sources and simplify queries across them.^11^

In order to explore the capabilities of semantic web technology, we have attempted to semantically link two disparate biological databases. The ALFRED database collects allele frequency values from a wide range of human populations.^12^,^13^ Starpath is a database of biological pathways and the individual interactions that comprise them.^14^ Both resources store data in traditional tables using a relational database management system (RDBMS). There is little else in common between these two databases. They were developed by different groups with different goals and using different strategies. To start, we needed to create an OWL ontology for each of the resources and then load existing relational data into RDF triples based upon this ontology. With these new triple stores in place, we were able to locate terms used by both ontologies and issue semantic queries using the common terms to bridge the two data stores. We used Oracle’s Semantic Technologies^15^ to perform these tasks within the context of an existing relational database. Using this approach, we were able to search for a number of genes involved in cancer pathways that show genetic variation within and among populations.

## Overview of Existing Technologies

**Figure 1:**
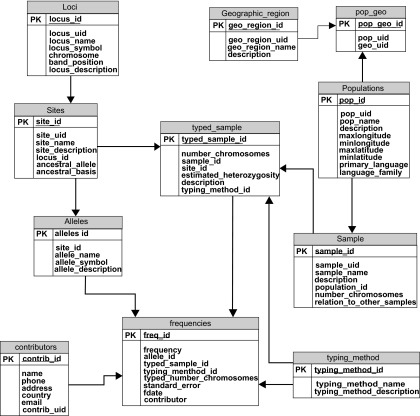
The ALFRED database schema.

ALFRED, the allele frequency database, provides allele frequency data for anthropologically defined human population samples.^16^ It contains both public data from literature and unpublished data from our host research laboratory and its collaborators. For data derived from literature, we tried to select those polymorphisms which have been studied in a wide variety of populations. ALFRED covers a broader spectrum of anthropologically defined populations than HapMap,^17^ another frequently sited source of allele frequency data. Over 95% of the polymorphisms in ALFRED have frequency data from more than 10 different populations. This is without considering the samples from different regions within the same population. We implemented ALFRED using a traditional relational structure which is illustrated in Figure 1. An individual polymorphism (or **Site**) is contained within a locus on the genome. Ethnic populations are organized by their geographic location (**Geographic_Region**). Multiple samples may be drawn from a particular population. For such highly heterogeneous populations as African American or European American, special care is taken to delineate the specific geographic region of the population. Population samples are typed to determine the frequency of alleles at a site. The **Typed_Sample** table bridges samples and polymorphisms and also associates the typing method, which is detailed in the **Typing_Method** table. The allele frequency values for a **Typed_Sample** are stored in the **Frequencies** table. Information about the contributor of particular allele frequency data is kept in the **Contributors** Table.

The ALFRED project is part of an international effort organized by JBIC (Japan Biological Informatics Consortium) to provide a standardized object model for genome sequence variation data. In June 2005, the initial version of this model, represented in XML through the Polymorphism Markup language (PML1), was approved by the Object Management Group (OMG).^18^ In September of that year, discussions began to expand this model to include phenotype data and genotype to phenotype (G2P) mappings. This extended model, called PAGE-OM (Phenotype and Genotype Experiment Object Model) intends to provide a common framework for management of any DNA variation data, phenotype data or G2P experimental findings. Although the model is not officially expressed in a technology-specific form, such as an XML or database schema, an XML representation called PAGE Markup Language (PML2) is being developed. PAGE-OM is currently under consideration as an OMG standard (PAGE-OM 2008). ALFRED currently supports export of its data in PML1 format for those polymorphisms that have rs identifiers in the dbSNP database. The two PML domains that can be used to represent ALFRED data are the **SAMPLE** domain and the **GENOTYPE** domain. While populations and their samples are defined separately in the ALFRED database, in the PAGE model, a population and the multiple samples taken from it are all represented by a single ‘**panel**’ class. We achieve the proper relationship by nesting multiple sample ‘panels’ within a population ‘**panel**’. Polymorphism sites, alleles and frequencies are represented using PML’s **GENOTYPE** domain. **Site** corresponds to the ‘**genomic_polymorphism**’ class, alleles to the ‘**genomic_allele**’ class and frequencies to the ‘**genomic_allele_population_frequency**’ class.

Starpath is a collection of resources for research on biological pathways. The goals of the project are to integrate information on pathways from a wide variety of sources and to provide tools that allow this information to be easily browsed and analyzed, with primary focus on statistical methods and visualization. Starpath is a three-tier application, built using Java/J2EE technology, with an Oracle relational database at its core. The relational tier is mapped to an object graph on the application server using JPA/Hibernate.An Application Programming Interface (API) is provided through Enterprise Java Beans (EJB) to allow a presentation layer to interact with the database. The presentation tier is a rich client application using Java’s Swing user interface library that can be activated using Java Web Start technology. It is possible that in the future, a web interface may be provided as an alternative presentation layer. A Web Service interface to the remote API is also planned for the near future.

The contents of the Starpath database have been determined largely based upon the interests of database users. Our researchers have typically wanted to see what genes are highly or differentially expressed in certain pathways based upon the results of microarrays and other types of high throughput analysis. As a result, our database schema is somewhat “gene-centric” in its design. We denote what individual events comprise particular pathways and the biological and chemical entities that make up these events. Wherever possible, these entities are linked to one or more genes; for example, proteins and enzymes are linked to the genes which encode them. In turn, individual genes are linked to those “gene products” with which they are related; such products include microarray probes, SNPs and so forth. Starpath holds information on all pathways from KEGG,^19^ Biocarta,^20^ GenMAPP,^21^ Ricecyc^22^ and Cancer Cell Map^23^ where it applies to the following five organisms: human, mouse, rat, dog and rice. An automated build system was designed to keep Starpath up-to-date and to allow new sources to be easily incorporated. We have incorporated several new sources since inception and many more are planned for the near future. A parser for the BioPAX pathway exchange format^24^ has been developed as part of our build suite to facilitate this process.

## Data Conversion

The first step of our workflow was to convert each of the two datasets from their extant relational form into something that could be queried semantically. To do this, it was necessary to define a new ontology for each, extract data from each dataset and load it into the “N-Triples” format used by Oracle’s Semantic Technologies API. For the purposes of this paper, we simplified the overall data models of ALFRED and Starpath somewhat in order to conserve space. We selected the 10 pathways defined by Sloan-Kettering’s Cancer Cell Map^23^ project as being representative cancer-related pathways in humans. Exporting these into our simplified ontological model for Starpath yielded around 230,000 triples. Export of the entirety of ALFRED into its simplified ontology produced approximately 2.5 million triples.

Oracle releases from 10 g onward have included support for semantic data storage.^1^ Oracle’s RDF storage works by treating the triple store as an application within a running database instance. Essentially, individual RDF triples are stored as rows in a conventional database table. For this reason, the amount of storage is not limited by the size of main memory as it is with the majority of RDF storage engines. Additionally, Oracle’s triple store can take advantage of the scalability and cost-based performance optimizations of Oracle’s relational storage engine. As a side-effect, we can combine semantic queries with relational query constructs, since the triples are actually stored as Oracle objects in a conventional relational table. This has its advantages as it allows for certain types of query that are typically difficult using semantic query languages. Oracle Semantic Technologies also provides a powerful inferencing engine. The engine implements the full set of RDF/S inference rules and OWLPrime, an important subset of the OWL DL vocabulary. OWLPrime consists of around 50 rules selected from OWL DL that Oracle felt would sufficiently balance expressivity with efficient performance. Support for user-defined rules is also provided. Finally, the Oracle RDF inference engine includes mechanisms for analyzing ancillary information, such as semantic distance and proofs, about triples and for validating semantic data models. Benchmarks conducted by Oracle indicate that the performance of the inference engine scales linearly for datasets into the hundreds of millions of triples.^25^

Conversion of a data model from a relational structure to a semantic one can be a difficult process. We have found, however, that if there exists a class model such as one would design to represent the data in an object-oriented (OO) language such as Java, the transition is more direct. Much has been written about the “object-relational” disconnect, but the object-semantic disconnect seems less onerous. Typically, we can recast OO classes as OWL classes and fields of the classes as OWL properties. OWL subclasses and superclasses behave like their OO counterparts. OWL datatype properties would be analogous to primitive types such as *int* or *float* or standard library classes such as *String.* Properties that are composed of objects of other classes defined in our model would correspond with OWL object properties. OWL properties with cardinality greater than one would be modeled as collections such as *Lists* or *Sets.* The analogy is not entirely perfect; e.g. OWL classes support multiple inheritance while many OO languages, including Java do not and OWL’s support for transitive properties and sub-properties would require some fancy footwork in an OO language. However, for the common case, object modeling and semantic modeling match up fairly well. There are in fact tools that will generate stub Java classes from existing OWL ontologies.^26^ We found the Protege application^27^ to be quite helpful in generating OWL ontologies. We discuss specific features of the two ontologies below.

Our ontology for the ALFRED data (illustrated in Fig. 3) is based upon the subset of PML that ALFRED uses for data export. In most cases, the class names and properties are drawn directly from the elements and attributes defined in PML’s xsd schema. A notable exception is in the handling of populations and samples, a central feature of the ALFRED database. Whereas in the PML format, each of these are treated as **Panel** elements with differing attributes, for the ALFRED ontology, we created distinct **Population** and **Sample** classes. We also define a **sample** object property of multiple cardinality within the **Population** domain to express the fact that individual **Populations** can have more than one **sample**. The **Population** class is further specified by a unique **id** from the ALFRED database and by datatype properties for **ethnicity, geographicRegion, languageFamily** and **primaryLanguage**. A paragraph-length **description** of the **Population** is also provided. A **GeographicLocation** class is defined to hold latitudinal and longitudinal data for the **Population** and is specified as an object property bounded by the **Population** class. The **Sample** class also holds a unique **id** generated by the ALFRED database and datatype properties indicating **countUnit** and **size** for the sample. A brief **description** further details the procedure used to gather the sample. The second central feature of the ALFRED semantic store is the **GenomicPolymorphism** class. In addition to its database-derived **id**, this class acts as domain for datatype properties representing the **snpID** from dbSNP,^28^ the **validationStatus** and zero to many corresponding **geneIDs** from the NCBI’s Entrez database. Two other classes are defined for ranges of object properties on the **GenomicPolymorphism** class. The **GenomicAllele** class defines the one or more alleles in question by its database-generated **id**. The **ReferenceGenomicLocationInAssembly** class details the location of the polymorphism upon the chromosome by specifying the **strand**, **chromosomeName, start** and **end** of the sequence. Finally, we define a **GenomicAllelePopulation-Frequency** class to join information about the polymorphism with information about the population. Along with object properties pointing to the **genomicAllele** and the **sample**, there are datatype properties giving the frequency **value** and **count**.

Full conversion of the Starpath database into RDF would generate something on the order of hundreds of millions of triples. For the purposes of this paper, we wished to deal with a much smaller volume of data and so several decisions were made to create a simplified ontology. As mentioned earlier, we decided to look at only the 10 pathways defined by the Cancer Cell Map^23^ project as indicative of cancer pathways. We also decided not to include several of the table attributes which were of lesser significance to this endeavor, especially those directly related to database metadata such as timestamp, owner and versioning information. We limited traversal of links between objects to one level and disregarded some other associations between objects in an attempt to limit the breadth of data. For example, while we included all gene products linked to a gene of interest we did not perform the reverse join and collect all other genes connected to each gene product. Because, we already had an object-oriented graph of the Starpath data model that is used in the object-relational mapping layer of the application, it was relatively straightforward to generate OWL classes and properties.

**Figure 2:**
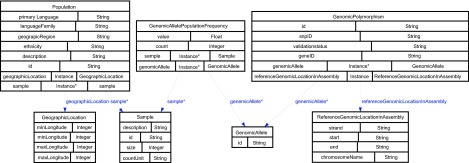
The ALFRED ontology. (Generated using Protege Ontoviz plugin). OWL classes are depicted in the boxes along with their properties. Object Properties are illustrated with edges connecting the class that owns the property with the class type of the property. The edge label is the name of the Object Property.

**Figure 3:**
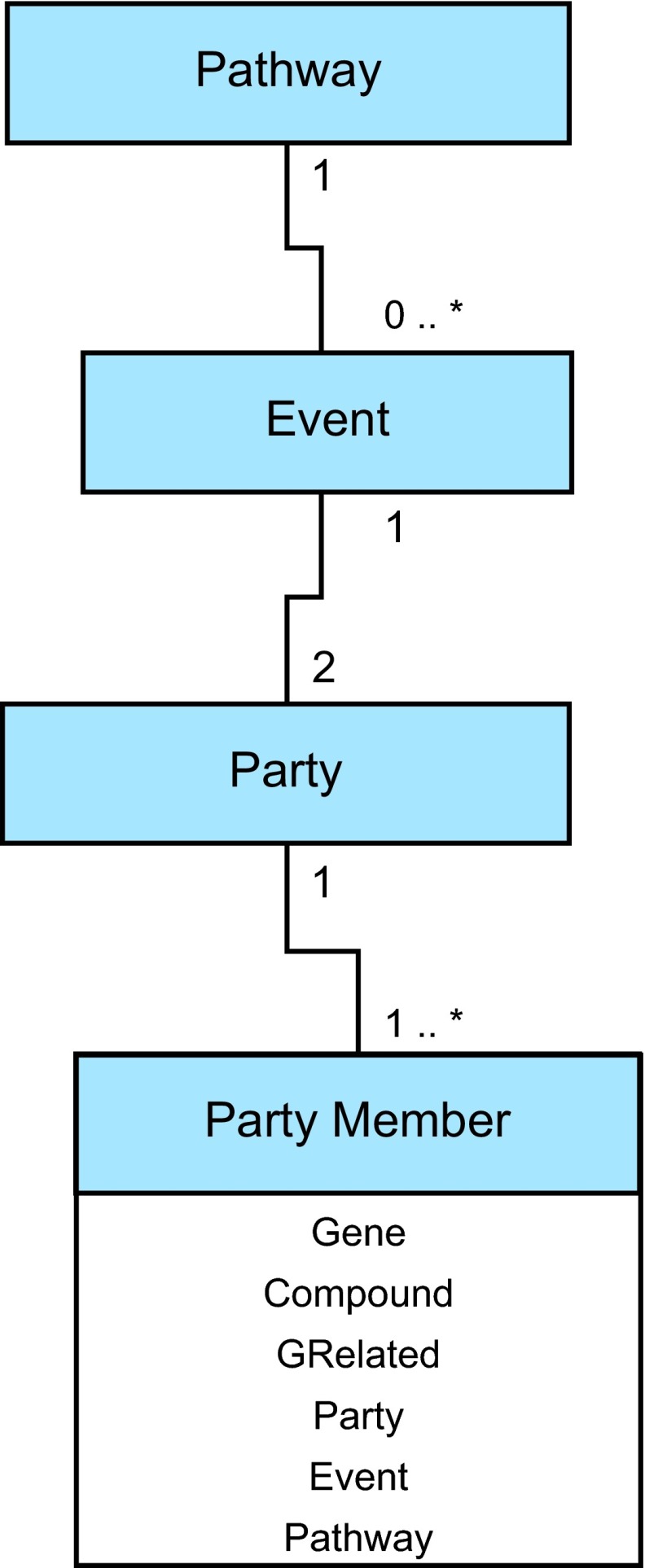
The core of the Starpath Pathway data model.

Starpath models pathways as networks of objects which can be nested to form a tree of arbitrary depth. The basic model is illustrated in Figure 2. The base unit is, of course, the **Pathway** which defines a collection of **Events**. Each **Event** is composed of two **Parties** arbitrarily designated as **Party1** and **Party2**. Each **Party,** in turn, is composed of one or more **PartyMembers**. These **Members** can be any of several types including **Genes; GRelateds,** which are other entities such as proteins, enzymes or microarray probes that can be linked to a gene; and **Compounds**, which are chemical or physical entities that are not associated with genes. **PartyMembers** can also be other **Parties**, **Events** or **Pathways**. Although there is no limit to the level or recursion that is allowed in a **Pathway** structure, circular object references have been eliminated. Each of these six components of the pathway tree is defined as subclasses of an abstract **SPElement** class which specifies properties common to each component. These are the **name** of the object, its **provenance** and its **ref-id** as designated within its originating source. **Provenance** is an object property whose range is a class called **Reference** which provides a **name** and **description** of the data source from which an **SPElement** derived.

**Figure 4:**
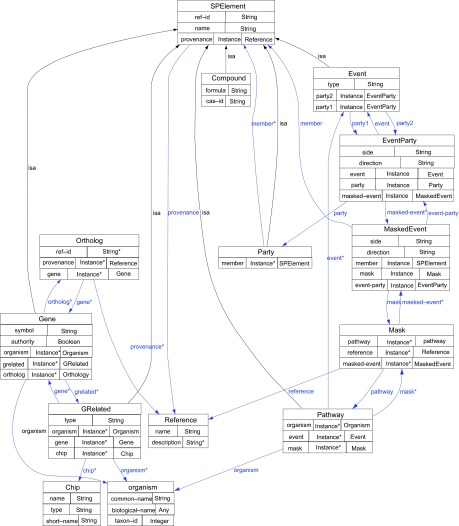
The Starpath ontology. (Generated using Protege—Ontoviz plugin).

Figure 4 shows the full Starpath ontology. Each of the **SPElement** subclasses defines a few custom properties. **Compounds** indicate their **cas-id** from the Chemical Abstract Services and their chemical **formula. Events** designate their **type** and use object properties to point to their constituent **party1** and **party2**. These object properties are **members** of a special class called **EventParty** which joins **Event** and **Party** and further specifies which **side** of the **Event** the **Party** occupies and in which **direction** the **Event** proceeds. The **EventParty** also holds references to **masked-events**. A **MaskedEvent** is an event as described by a pathway where only a portion of the event in question is significant to definition of the pathway. For example, though by definition any biochemical reaction is reversible given the necessary conditions, often the reaction will only proceed in one direction within a metabolic pathway. An instance of the **Mask** class is used to indicate given a certain **pathway** from a particular **reference** what **masked-events** are in effect. The **MaskedEvent** class in turn indicates given the **event-party** and **mask**, which **members** will participate and on which **side** and **direction**. The **Gene** class holds datatype properties that provide the common **symbol** for a gene and whether this symbol is governed by a particular **authority** such as HUGO for human genes. Gene also specifies zero to many **grelated** objects that are associated with it and zero to many **orthologs. Orthologs** are described by the **Orthology** class which delineates their **provenance** through a **Reference** object and their **ref-id** from that **provenance**. The **organism** of a gene is an instance of an **Organism** class, which provides its **biological-name, common-name** and **taxon-id**. As mentioned above, the **GRelated** class can describe any of several objects that can be linked to a gene. Its actual **type** is indicated by datatype property and its organism by the **organism** object property defined for **Gene**. A common type of **GRelated** in Starpath is the microarray probe; for **GRelateds** of this type, the parent chip is specified in an object property whose range is the **Chip** class. The **Chip** class indicates its **full name, short-name** and **type** using datatype properties. The **Party** class adds no additional properties to its base class other than zero to many **members** whose range is any subclass of **SPElement**. Finally, we have the **Pathway** class, which contains object properties that list the **events** that comprise them, the **organism** in question and what **mask** if any is to be applied.

With our ontologies in place, we move to the next stage, the conversion of data to the new model. This is done in two phases. First, the existing data is loaded into an object graph made up of stub classes generated from the ontology. Secondly, the Java object graph is converted into an RDF graph which follows our ontology. If the data is stored in a database, the relational to Java stage is handled by calling a stored procedure through JDBC. This procedure returns arrays of Oracle records which correspond to our ontology classes; for simpler cases we may need only to return rows from particular tables. For data in another XML format, we populate the object graph by using a DOM parser such as Dom4j.^29^ The object to RDF phase performs two passes over the object graph. In the first pass, RDF nodes are created for each instance of each class in the ontology. The Data Properties are filled in with their literal values. At this point, we can’t resolve the Object Properties to RDF nodes because not all of the RDF nodes have been created. Therefore, on the first pass, we store pointers parsed nodes in a hash table. On the second pass, we use this hash table to resolve the Object Properties to existing RDF nodes. We use Hewlett Packard’s open source Jena Semantic Web framework^30^ to generate the RDF files. The entire process requires writing quite a lot of code, most of it repetitive and verbose. We hope to create a framework for generating a lot of this code using some type of mapping file in the near future. A SPARQL endpoint for the datasets that were generated for this project as well as the complete set of RDF triples and OWL ontologies are available at “http://bioinformatics.med.yale.edu/sparql/spalfred”.

## Example Queries

Having created ontological models for our two domains and populated them with relevant data, our next task is to determine what meaningful semantic queries can be performed. A number of languages for querying semantic data have been created over the years. Recently, SPARQL has emerged as a clear leader, having been standardized by the World Wide Web Consortium.^31^ The Oracle Semantic Web interface supports a subset of SPARQL functionality. For example, it lacks implementations of OPTIONAL and UNION clauses. These absences are partially mitigated, however, by the ability to apply traditional SQL to the table returned by the semantic query. Like SPARQL queries, Oracle’s semantic queries are made up of sequences of triple patterns of the form (*subject predicate object*). Variables preceded by a ‘*?*’ can be substituted for any of these terms; matching triples will bind actual values to the placeholder variables. Returned are those results that satisfy all of the triple patterns in the sequence. In the Oracle Semantic Technologies API, this is implemented as a call to a stored procedure and the results can be returned in a conventional table. Oracle also offers support for user-defined rules through which we can state that given a set of antecedent triple patterns, a consequent triple pattern can be inferred.^25^ A simple example is the ‘grandfather’ rule: given *(A father Of B) (B father of C)* it can be inferred that *(A grandfather Of C).* Custom rules are useful for a number of reasons. They can simplify complicated logical relationships and allow for cleaner and more intuitive queries. Additionally, we can create entailments in which all results of a rule are pre-calculated and indexed. This can provide significant performance improvements.

One way in which semantic queries are particularly helpful for exploring Starpath data is that they can flatten the tree structure to find elements at any of multiple levels. Suppose that we are searching within a pathway for a particular gene that is linked to a protein that is part of a complex of proteins that is part of an enzyme that catalyzes a particular reaction. In the terms of Starpath’s data model, this would involve five joins: **Pathway-Event** (the catalysis); **Event-Party** (the enzyme); **Party-PartyMember** (the protein complex); **Party-PartyMember** (the individual protein); and **Grelated** (the protein)**-Gene**. Because the **Party-PartyMember** join can be an arbitrary number of levels deep, we cannot fully resolve it with conventional SQL. Two possible solutions are the use of database-specific hierarchical query features such as Oracle’s *START WITH* and *CONNECT BY* or use of a stored procedure to walk the full extent of the tree. We use the second option for Starpath, because the **Party-PartyMember** join behaves differently depending on the type of **Party Member** with which we are dealing. By defining a handful of rules and creating entailments upon them we can perform this type of query on any SPARQL implementation or “SPARQL-ish” syntax like that of Oracle’s Semantic Web APIs. First we create a consequent property called ‘**memberOfParty**’ which is defined by two rules. **MEM_PARTY_RULE1** states that if *(?Xrdf: type :Party) (?X #member ?Y)* then *(?Y #memberOfParty ?X).* This is fairly obvious in that if X is of class **Party** any **member** property of it can also be called a **memberOfParty** of X. In **MEM_ PARTY_RULE2** (if *(?X #memberOfParty ?Y) (? X#memberOfParty ?Z)* then *(?Y #memberOfParty ?Z))*, we further specify that if any **member** of a **Party** is also a **Party** itself then that **Party’s members** are also **members** of the initial **Party**. This in effect flattens the hierarchy tree when it is run to closure. We create a consequent property called ‘**memberOfEvent**’ which moves up the hierarchy to state that **members** of **Parties** are also **members** of the **Events** to which the **Parties** belong. It requires two rules: a. if *(? X#memberOfParty ?Y) (? Z #partyl ?Y)* then *(?Y memberOfEvent ?X)* and b. if *(?X memberOfParty ?Y) (?Z #party2 ?Y)* then *(?Y #memberOfEvent ?X).* Finally ‘**memberOfPathway**’ is defined by stating that if *(?X memberOfEvent ?Y) (?Z #event ?Y) (?Z rdf:type #Pathway)* then *(?X #memberOfPathway ?Z).* This simply means that **members** of **Events** are also **members** of the **Pathways** the **Events** comprise. Now, we can retrieve a list of all genes that are involved in a particular pathways by asking *(?X rdf:type :Gene) (?X #memberOfPathway ?Y) (?Y :ref-id “InterestingPathway”).* We can use these custom rules to define further rules as well. Here is a possible rule to define two genes as “neighbors”, i.e. participants in the same **Event** in the same **Pathway**:if*(?GENE1:memberOfEvent ?EV) (?GENE1 rdf:type :Gene) (?GENE2 :memberOfEvent ? EV)(?GENE2 rdftype :Gene) (?PW :event ?EV) (?PW rdf:type :Pathway)* then *(?GENE1 #neighboringGene ? GENE2)*.

For the purpose of our analysis, the queries on the ALFRED semantic store are comparatively straightforward. We need to collect all frequency values from all samples that have been typed for a specific polymorphism of interest. Because none of the relations between these concepts can form multi-level hierarchies, we need not use recursion to reach closure. Creating additional entailments does not offer us any advantages and we can get results through a conventional join query. One possible form is the following sequence of triple patterns: “*(? poly rdf:type alf:GenomicPolymorphism) (?poly alf:genelD ?ref_id) (?poly alf:genomic Allele ?allele) (?poly alf:id ?poly_id) (?allele alf: id ?allele_id) (?gapf rdf:type alf:GenomicAllelePopulationFrequency) (?gapf alf:genomicAllele ?allele) (?gapf alf:value ?value) (?gapf alf:sample ?sample) (?pop rdf:type alf:Population) (?pop alf: sample ?sample) (?pop alf:ethnicity ?eth).*”

Now that our queries are in place on both stores separately, we need to perform the semantic link to obtain population data on polymorphisms that are also part of significant cancer pathways. Oracle Semantic Technologies makes this easy by allowing us to query across multiple semantic models within the database. We use the *‘alf’* prefix to reference the ALFRED ontology and the *‘sp’* prefix to reference the Starpath ontology. Our final query looks like this: “*(?gene rdf:type sp:Gene) (?gene sp:ref-id ?ref_id) (?gene sp:symbol ?symbol) (? poly rdf:type alf:GenomicPolymorphism) (?poly alf:genelD ?ref_ id) (?poly alf:genomicAllele ?allele) (?poly alf:id ?poly_id) (?allele alf:id ?allele_id) (?gapf rdf:type alf:GenomicAllelePopulationFrequency) (?gapf alf:genomicAllele ?allele) (?gapf alf:value ?value) (?gapf alf:sample ?sample) (?pop rdf:type alf: Population) (? pop alf:sample ? sample) (?pop alfethnicity ?eth).*” One possible optimization we can perform is to create an entailment that computes which polymorphisms we are retrieving from ALFRED based upon whether they are derived from genes which are also members of the Starpath pathways of interest. The following rule would be applied: “if *(?gene rdf:type sp:Gene) (?gene sp: ref-id ?ref_id) (?poly rdf:type alf:GenomicPolymorphism) (?poly alf:genelD ?ref_id)* then *(?poly alf: inStarpath “T”).*” We may now filter out polymorphisms which are in Starpath pathways with one triple: “*(?poly alf:inStarpath “T”).*”

## Discussion

The work presented in this paper can be considered an example of what is known in web circles as a “mashup”. In a typical mashup, divergent data are joined in a comparatively loose fashion to provide new understanding that cannot be attained from the data sources separately. Instead of aiming for total integration of disparate data sources, developers of mashups typically try to use as light-weight an approach as possible. The focus is on integrating data sources only as much as is needed for the task at hand. This allows for rapid development and promotes loose coupling of data. A semantic mashup (or “smashup”) is simply a mashup where the data are joined in a semantic fashion, i.e through RDF, OWL or similar technologies. Examples of this approach are starting to emerge in the field of biomedical science.^32^ Tools like TAMBIS^33^ and BirnLex^33^ provide broad frameworks through which data sources can be combined and queried. At a recent meeting of the World Wide Web Consortium’s Health Care and Life Sciences Interest Group, a demonstration was created that mashed images from the brain with data from the Entrez database and the Gene Ontology.^35^ Others, such as Sahoo et al.^36^ and Villanueva-Rosales and Dumontier^37^ use semantic mashup techniques to create knowledge bases focusing upon specialized areas of interest. Our work is similar to that of these last in that it focuses on tackling an immediate problem. Although aspects of our approach may be applied to semantic mashups in general, we do not propose a universal solution for merging all divergent biomedical data sources. We hope that other researchers may adapt some aspects of our methodology while modifying the rest to suit their individual needs. One distinction in our approach is that we define our own ad-hoc ontologies for the purpose of the mashup. This is possible because we control both of the data sources that are being merged. This allows us to link the data stores without many of the difficulties in joining heterogeneous ontologies. Of course this involves additional effort in the design stage of the project but it makes the interactions between data sources cleaner and more loosely coupled. Another issue is potential lack of portability. In the future it may become desirable to write code to translate our data from the custom ontology to a more universal format.

Having integrated pathway and allele frequency data presents us with a number of possibilities for analysis. As a proof of concept demonstration, we performed the relatively simple FST calculation^38^ on the 488 polymorphisms that were linked between ALFRED and Starpath. The FST calculation measures the proportion of total genetic variance within a sub-population to the total genetic variance.^39^, ^40^ Other more involved methods are possible.^41^ It is important to recognize also that because our knowledge of pathways is still nascent, a large number of genes known to be involved in tumorigenesis have not yet been associated with any particular cancer-related pathways. By the same token, there are also genes for which ALFRED does not have polymorphism data at present. Locating and incorporating high throughput genotype and allele frequency datasets from whole genome association studies is a key area of focus for the ALFRED project. One of the first dataset we uploaded was the allele frequency data for 11,555 SNPs typed on 12 population samples using whole genome sampling analysis (WGSA) technology.^42^ The next set we are interested in is the 650,000 Illumina-assayed SNPs typed on the HGDP-CEPH Human Genome Diversity Cell Line Panel which covers 51 different populations.^43^ The infusion of this new population data combined with advancing knowledge of cancer pathway mechanisms should greatly increase the effectiveness of our analyses.

A common issue in linking multiple semantic stores is the resolution of common terms. Biology and medicine are fields in which many equivalent terms are in circulation. For this paper, the common term in the data stores is the Entrez database identifier for a gene. This was easy to determine because we are knowledgeable about both data stores. Resolution can be much more difficult if we are dealing with more complicated links or with semantic data whose formats or even domains are unfamiliar to us. In such cases, controlled vocabularies are essential. The NCI Thesaurus and its parent the UMLS^44^ are important examples for the domain of cancer research. Ontologies that conform to these controlled vocabularies can communicate with each other without fear of meaning being lost through use of synonymous terms. For example, caBIG uses NCI Thesaurus terms as the basis for semCDI,^45^ which provides a common syntax for querying multiple data sources. As we further our exploration of linkage between pathway and genotype data for the purposes of cancer research, especially if we wish to integrate our data with caBIG, it will become useful to employ NCI Thesaurus terms in our ontologies.

For the queries we have performed both data sources are co-located within the same instance of an Oracle database. This will not always be convenient or even possible when performing queries across multiple semantic stores. Constraints of time and space often limit fully incorporating entire data sources locally. For this reason, Web Services, which provide access to remote data through a platform-independent XML-based format, have become increasing prevalent in medical informatics. For example, the Pathway Commons project provides web service access to information on their collection of biological pathways^46^ and the caBIG project provides a Web Services interface to its bioinformatics grid.^47^ Emerging standards such as OWL-S allow remote ontologies to be discovered and queried through Web Services.^48^ Starpath currently provides EJB access to its database for its rich client platform, but Web Service access to the same API is planned for the near future. We also hope to expose ALFRED’s data to a Web Service soon.

Comparisons will naturally be drawn between the Starpath ontology and that of the BioPAX project, since both use OWL to model the domain of biological pathways. Our ontology differs in part because it is derived from databases which preceded the standardization of BioPAX Level 1. Despite this, we have closely followed BioPAX’s growth and it has influenced the development of our data model. A key difference in strategy is that whereas BioPAX strives for rigorous definition of pathway mechanics, our central focus is on a format sufficiently generic to incorporate a variety of conceptions of pathways. More precise definition can be supplied through various descriptive properties such as the Event class’s type property. The trade-off of course is that it is more difficult to enforce structural rules with our model. The Starpath model can currently support any sort of pathway, including many not currently supported in BioPAX. For example, support of gene expression is currently being developed as part of BioPAX level 3. Ability to interact with the BioPAX specification is a key goal of the Starpath project. We have developed a BioPAX parser that is used to import a variety of pathways including the Cancer Cell Map 22 pathways that we examined for this paper. We plan to create a utility to export our pathways into BioPAX format in the near future.

The extended version of PAGE-OM discussed above incorporates both genotype and phenotype modeling. While ALFRED does not hold phenotype data, the PAGE-OM model can be used to represent information by combining two different but related databases, one holding genetic variation data and other disease related data. We feel that in this way, the PAGE-OM schema could potentially be used to represent Starpath-ALFRED integrated data. The ‘**Observable_features**’ class is given a recursive association with itself, allowing related phenotypes to be nested. For instance, an **Observable_features** instance “Type II Diabetes Disease Status” may be assigned other instances “body-mass-index” and “Glucose tolerance”. The **EXPERIMENT** domain is used to unite the **PHENOTYPE** and **GENOTYPE** domains. The ‘**Experiment_result**’ class in the **EXPERIMENT** domain is provided with associations to classes such as ‘**Observable_feature**’, ‘**Observed_value**’ (the phenotype measurement being considered), ‘**Genomic_variation**’ (the marker examined) and ‘**Genomic_observation**’ (the genotype measurement).

Semantic Web technology plays a central role in the future plans of the Starpath project. Work is already under way to expand our ontology to cover the entirety of the Starpath data model and to convert increasing amounts of our data into RDF triples. As we do this, we hope to make our data available to public inquiry through Semantic Web Services and possibly through integration with caBIG. We intend for the Starpath ontology to serve as the central data model for our entire project. We are working on ways of directly tying our database schema and object-relational model directly to the OWL ontology through the development of code generation utilities.

## Conclusion

Semantic Web technologies offer powerful new ways to integrate data from disparate sources. They also provide us with meaningful new ways to query this data. We have explored some of the issues involved with the semantic linking of pathway data with population-based allele frequency data. We have also illustrated examples of interesting queries that can be performed upon this linked data. Finally, we have presented some potential future benefits that can be derived from the combining of pathway and population genetics data and from the use of semantic technologies in this area of cancer research.
